# Assessment of Reticulocyte and Erythrocyte Parameters From Automated Blood Counts in Vaso-Occlusive Crisis on Sickle Cell Disease

**DOI:** 10.3389/fmed.2022.858911

**Published:** 2022-04-13

**Authors:** Guillaume Feugray, Fiston Kasonga, Maximilien Grall, Ygal Benhamou, Victor Bobée-Schneider, Gérard Buchonnet, Sylvie Daliphard, Véronique Le Cam Duchez, Agnès Lahary, Paul Billoir

**Affiliations:** ^1^General Biochemistry, Normandie Univ, Rouen University, INSERM U1096, CHU Rouen, Rouen, France; ^2^CHU Rouen, Vascular Hemostasis Unit, Rouen, France; ^3^Department of Internal Medecine, CHU Rouen, Rouen, France; ^4^Department of Internal Medecine, Normandie Univ, Rouen University, INSERM U1096, CHU Rouen, Rouen, France; ^5^CHU Rouen, Hematology Laboratory, Rouen, France; ^6^Normandie Univ, Rouen University, INSERM U1096, CHU Rouen, Vascular Hemostasis Unit, Rouen, France

**Keywords:** sickle cell disease, vaso-occlusive crisis, reticulocyte count, erythrocyte parameters, immature reticulocyte fraction

## Abstract

Sickle cell disease is a complex genetic disease involving cell adhesion between red blood cells, white blood cells, platelets and endothelial cells, inducing painful vaso-occlusive crisis (VOC). We assessed reticulocyte and erythrocyte parameters in a cohort of confirmed SCD patients, and investigated whether a combination of these routine laboratory biomarkers of haemolysis could be used to predict VOC development. Reticulocyte and erythrocyte parameters were evaluated using the Sysmex XN-9000 analyser. A total of 98 patients with SCD were included, 72 in steady state and 26 in VOC. Among the 72 patients in steady state, 22 developed a VOC in the following year (median: 3 months [2–6]). The following parameters were increased in SCD patients with VOC development compared to SCD patients without VOC development in the following year: reticulocyte count (94.6 10^9^/L [67.8–128] vs. 48.4 10^9^/L [24.9–87.5]), immature reticulocyte count (259 10^9^/L [181–334] vs. 152 10^9^/L [129–208]) reticulocyte/immature reticulocyte fraction (IRF) ratio (6.63 10^9^/(L^*^%) [4.67–9.56] vs. 4.94 10^9^/(L^*^%) [3.96–6.61]), and medium fluorescence reticulocytes (MFR) (19.9% [17.4–20.7] vs. 17.1% [15.95–19.75]). The association of a reticulocyte count of >189.4 10^9^/L and an MFR of >19.75% showed a sensitivity of 81.8% and a specificity of 88% to predict VOC development in the following year. Based on our findings, a combination of routine laboratory biomarkers, as reticulocyte count, immature reticulocyte count and fluorescent reticulocyte fraction at steady state, could be used to predict VOC development in SCD.

## Introduction

Sickle cell disease (SCD) is an inherited haemoglobinopathy disorder caused by mutations in *HBB* gene with amino-acid substitution on β globin chain. The consequence is synthesis of altered hemoglobin S (HbS) which polymerises in red blood cell (RBC) at deoxygenated state. SCD is associated with chronic haemolytic anemia, vaso-occlusive crisis (VOC) leading to frequent hospitalization, morbidity and mortality caused by organ failure like stroke, acute chest syndrome (ACS), osteonecrosis, leg ulcers, retinopathy, pulmonary hypertension, priapism and nephropathy (1). Moreover, hypercoagulability state is reported in SCD with increased venous thromboembolism (VTE) or pulmonary embolism (PE) (2).

Automated hematology analysers provide whole blood counts with increasing reliability and accuracy (3). However, in daily laboratory practice, no analyser can determine properly RBC morphological abnormalities. These criteria are not always relevant hence the interest to combine several RBC and reticulocyte parameters in order to improve the specificity and sensitivity of microscope examination of a blood smear (4). The introduction of additional parameters in the past years has opened new opportunities for red cell evaluation. The evaluation of automatic parameters of reticulocyte fraction has been reported to be useful in the screening of haemolytic anemia. In Sysmex analysers, reticulocytes are fractionated in low (LFR), medium (MFR) and high fluorescence reticulocytes (HFR). Reticulocytes can be classified according to RNA content into subtypes reflecting successive stages during maturation: immature with HFR to MFR and mature with LFR (5). Whole reticulocyte fraction provides information on bone marrow recovery. However, few studies have evaluated these parameters in sickle cell disease and VOC development (3, 4, 6).

The aim of the study was to assess reticulocyte and erythrocyte parameters in a cohort of confirmed SCD patients, and to investigate whether a combination of these routine laboratory biomarkers of haemolysis could be used to predict VOC development.

## Methods

### Study Design and Patients

All patients in the study were diagnosed and treated for SCD at Rouen University Hospital between September 2018 and June 2021. Patients were included during injury evaluation or VOC hospitalization in our tertiary center. The injury evaluation was a standard of care, every year. Patients with VOC were included <24 h after admission to emergency department. All patients received a systematic annual consultation for injury evaluation and determined VOC development.

Prospective data were collected and completed from medical records. Clinical information included age and sex. Pregnant women and patients <18 years were excluded.

The study was performed in accordance with the Declaration of Helsinki on biomedical research involving human subjects. The institutional review board (Rouen University Hospital) approved the study (Authorization protocol number: E2021-78).

### Samples and Analysis

Biological standard follow-up included sampling of dipotassium EDTA tubes (BD Vacutainer EDTA, Plymouth) for whole blood counts and plasma from lithium heparin tubes with gel separator (BD Vacutainer LH, Plymouth) for biochemical parameters.

The severity of haemolysis was estimated using plasma lactate dehydrogenase level (LDH). These tests were performed in samples collected for routine follow-up. Whole blood counts were measured on XN-9000 (Sysmex, Villepinte, France). LDH, creatinine, ferritin and indirect bilirubin levels were determined on cobas^®^ 8000 chemistry analyser (Roche Diagnostics, Mannheim, Germany).

### Reticulocyte and Erythrocyte Measurement

RBC count was measured using the impedance variation method after hydrodynamic focusing. SCD samples were analyzed in the RET (reticulocytes) channel and classified as per the cell blood count optic (CBC-O) application developed by Sysmex™ that is embedded in the Extended Information Processing Unit (Extended-IPU) (7). Reticulocyte measurement was based on the principle of fluorescence flow cytometry using the nucleic acid dye oxazine 750 which stains the RNA of the cell (8). According to their stage of maturity, reticulocytes have varying fluorescence intensity, and accordingly, they are divided into three subtypes: mature reticulocytes with limited fluorescence are called low-fluorescence reticulocytes (LFR), intermediately mature reticulocytes with medium fluorescence are called medium-fluorescence reticulocytes (MFR), and very immature reticulocytes with high fluorescence are called high-fluorescence reticulocytes (HFR). The immature reticulocyte fraction (IRF), is the percentage of immature reticulocytes calculated as the sum of MFR and HFR and is a reflection of erythropoietic activity (9). Analysis were performed before any transfusion who potentially change results. Analysis were performed only one time in patients.

Parameters of interest provided by reticulocyte and erythrocyte measurement are (10):

- Hemoglobin (Hb), Mean corpuscular volume (MCV), mean corpuscular hemoglobin concentration (MCHC).
- Immature red cells: Reticulocytes, IRF, LFR, MFR, HFR, hemoglobin concentration in reticulocytes (Ret-He), part of young reticulocytes (reticulocytes/IRF).
- Hypo-He and Hyper-He corresponding to red blood wwwcells with hypo-hemoglobin content (<17 pg) and hyper-hemoglobin content (>49 pg), respectively.
- The percentage of microcytic erythrocytes (%Micro-R) and macrocytic erythrocytes (%Macro-R).

### Statistical Analysis

Data are expressed as median and interquartile ranges [IQR]. Statistical analyses were performed with GraphPad Prism for Windows, version 9.2. (GraphPad Software, San Diego, California, United States). Fisher exact test, Kruskall–Wallis ANOVA with Dunn's multiple comparisons post-test, Mann-Whitney test or Chi square test were used. Receiver operating characteristic (ROC) curves were built for significant clinical characteristics. *P* < 0.05 were considered to be statistically significant.

## Results

### Demographic Characteristics

A total of 98 patients with SCD were included in this study, 72 in steady state and 26 during VOC. Demographic and clinical characteristics are reported in Table 1. Forty-three patients had homozygous SCD (S/S) or heterozygous SCD and β^0^ thalassaemia (S/β^0^), 26 had homozygous SCD with α^3.7^ thalassaemia (S/Sα^3.7^), 29 had heterozygous SCD with C hemoglobin (S/C) or β^+^ thalassaemia (S/β^+^). All genotypes were represented in VOC. In the 72 patients in steady state, 22 developed a VOC during the following year (median: 3 months [2–6]).

**Table 1:** Characteristics of study population.

	**SCD at steady state**	**SCD in VOC**	***P***
	***n* = 72**	***n* = 26**	
**Clinical characteristics**
Age (years)	35.1 ± 12.5	30.7 ± 9.3	0.067
Male *n* (%)	31 (43)	12 (46)	0.82
Hydroxyurea *n* (%)	39 (54)	19 (73)	0.11
Osteonecrosis *n* (%)	23 (32)	3 (11.5)	0.067
Retinopathy *n* (%)	13 (18)	9 (34.6)	0.10
Vasculopathy *n* (%)	9 (12.5)	5 (19.2)	0.51
ACS *n* (%)	20 (27.7)	7 (26.9)	>0.99
Cholecystectomy *n* (%)	28 (38.9)	7 (26.9)	0.34
Splenectomy *n* (%)	2 (2.8)	4 (15.3)	**0.04**
**Hematological parameters**
RBC (10^12^/L)	3.13 [2.67–3.97]	2.73 [2.39–3.18]	**0.017**
Hemoglobin (g/dL)	9.25 [8–10.5]	8.8 [8–10.1]	0.41
Haematocrit (%)	28 [23–30.7]	25 [23–29]	0.28
MCV (fL)	82 [73.6–91.1]	91.7 [79.8–101.3]	**0.026**
MCHC (g/dL)	34.7 [33.0–35.7]	35.1 [34.3–36.0]	0.08
Platelets (10^9^/L)	308 [180–391]	328 [258–451]	0.11
Leukocytes (10^9^/L)	7.1 [5.73–9.78]	10.7 [7.7–12.5]	**<0.001**
Neutrophils (10^9^/L)	3.94 [2.90–5.64]	5.60 [3.99–7.71]	**0.0033**
Lymphocytes (10^9^/L)	2.23 [1.53–2.98]	2.68 [2.16–3.59]	**0.014**
Monocytes (10^9^/L)	0.69 [0.48–1.00]	0.89 [0.59–1.31]	**0.0048**
HbF (%)	4.9 [2.6–11.3]	11.1 [2.5–17.6]	0.36
**Biochemistry parameters**
Indirect bilirubin (μmol/L)	9 [8-11]	10 [8-11]	0.72
LDH (U/L)	347 [230–448]	406 [345–499]	**0.046**
Ferritin (μg/L)	135 [52–282]	83 [58–108]	0.59
Creatinine (μmol/L)	59 [53-78]	49 [41-61]	**0.0065**

*Data are expressed as median and [IQR] except for age (mean ± SD) and clinical characteristics with n is the total number of patients (%), ACS, acute chest syndrome; HbF, hemoglobin F; RBC, red blood cells; MCV, mean corpuscular volume; MCHC, mean corpuscular hemoglobin concentration; LDH, lactate deshydrogenase. Bold values represent significant differences between the groups*.

We observed more splenectomy in the VOC group (*p* = 0.04) and significant differences between patients included during VOC and patients included in steady state, a decrease of RBC (*p* = 0.017), and an increase of MCV (*p* = 0.026). In whole blood count, we observed during VOC, significant increases of leukocytes (*p* < 0.001), neutrophils (*p* = 0.0033), lymphocytes (*p* = 0.014) and monocytes (*p* = 0.0048). Moreover, in biochemistry parameters, in the VOC group, LDH was significantly increased (*p* = 0.046) and creatinine significantly decreased (*p* = 0.0065).

### Reticulocyte and Erythrocyte Variations During VOC

Reticulocyte and erythrocyte parameters are reported in Table 2. Briefly, SCD patients with VOC had significantly higher reticulocyte counts (*p* = 0.0073), higher immature reticulocyte ratio (reticulocytes/RET-IRF, *p* = 0.004) and increased Hyper-He (*p* = 0.047). No other parameters had significant differences.

**Table 2:** Reticulocyte and erythrocyte parameter comparison between steady state vs. VOC.

**Parameters**	**Steady state**	**VOC**	***p* value**
	***n* = 72**	***n* = 26**	
Reticulocytes (10^9^/L)	184 [138–286]	276 [181–385]	**0.0073**
Reticulocytes (%)	6.06 [3.2–10.0]	9.62 [6.05–13.7]	**0.0038**
RET-IRF (%)	35.1 [28.7–40.6]	35.3 [26.6–42.9]	0.94
RET-IRF value (10^9^/L)	65.9 [40.4–113.4]	87 [52.6–147.4]	0.056
RET-He (pg)	31.5 [28.2–34.3]	32.9 [29.9–35.2]	0.31
Reticulocytes/RET-IRF (10^9^/(L*%))	5.52 [4.09–7.4]	7.77 [5.04–10.5]	**0.004**
Hypo-He (%)	5.2 [1.8–12.3]	1.8 [1.1–5.5]	0.053
Hyper-He (%)	0.50 [0.30–0.80]	0.80 [0.40–3.2]	**0.047**
Micro-R (%)	13.6 [5.25–26.5]	7.4 [3.0–16.2]	0.052
Macro-R (%)	3.9 [3.2–6.0]	5.1 [3.7–8.8]	0.061
LFR (%)	65.3 [59.6–71.5]	66.1 [59.6–71.0]	0.77
MFR (%)	18.0 [16.1–20.1]	18.1 [16.8–19.9]	0.94
HFR (%)	17.1 [11.8–20.8]	15.5 [11.7–21.2]	0.62

*Data are expressed as median ± [IQR], n is the total number of patients. LFR: low fluorescent reticulocyte. MFR, Medium fluorescent reticulocyte; HFR, High fluorescent reticulocyte; VOC, Vaso-occlusive crisis. Bold values represent significant differences between the groups*.

### Reticulocyte and Erythrocyte Variations in Patients Developing VOC

**Figure 1:**
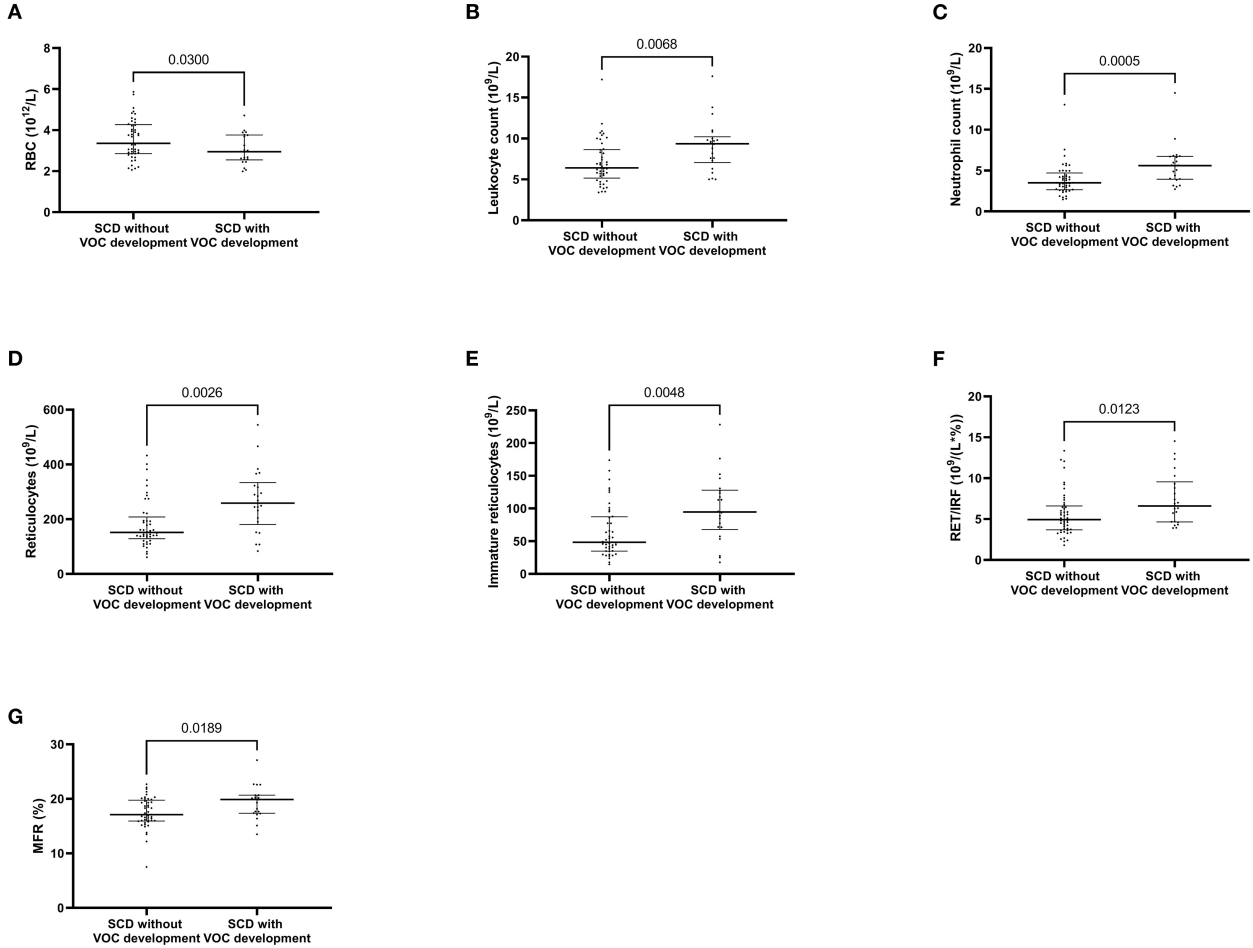
Whole blood count and reticulocyte parameters associated with vaso-occlusive crisis development. Red blood cells **(A)** Leukocyte count **(B)** Neutrophil count **(C)** Reticulocytes **(D)** absolute value of immature reticulocytes **(E)** Part of immature reticulocyte fraction **(F)** medium fluorescent reticulocytes **(G)**. *P* values comparing clinical improvement to clinical worsening are from Mann-Whitney *U*-test.

We determined which SCD patients included at steady state were hospitalized for VOC (*n* = 22) in the following year. Results are reported in Table 3 and in Supplemental Table 1 between genotype. Genotype was not associated with VOC development (Chi Square, *p* = 0.08). In patients with VOC development, RBC were significantly decreased (*p* = 0.03), leukocytes and neutrophils were increased (*p* = 0.0068 and *p* = 0.005, respectively) (Figure 1). Moreover, significant increases were observed for reticulocyte count (*p* = 0.0026), immature reticulocyte count (*p* = 0.0048), reticulocytes/IRF ratio (*p* = 0.018) and MFR (*p* = 0.0189).

**Table 3:** Comparison of biologic parameters in SCD patients developing or not a VOC in the following year.

	**SCD without VOC development *n* = 50**	**SCD with VOC development *n* = 22**	***P***
RBC (10^12^/L)	3.36 [2.86–4.27]	2.95 [2.55–3.77]	**0.030**
Hemoglobin (g/dL)	9.8 [8.15–10.58]	8.4 [7.75-10.25]	0.12
Haematocrit (%)	28 [23.5–31]	24 [22–29.3]	0.087
MCV (fL)	76.3 [72.1-89.2]	83.6 [79.7-95.0]	0.082
MCHC (g/dL)	34.6 [32.9–35.7]	35.0 [33.6–35.6]	0.49
Platelets (10^9^/L)	302 [168.5–382]	352.5 [238.5–434]	0.083
Leukocytes (10^9^/L)	6.4 [5.15–8.65]	9.35 [7.05–10.2]	**0.0068**
Neutrophils (10^9^/L)	3.51 [2.66–4.72]	5.61 [3.94–6.74]	**0.005**
Lymphocytes (10^9^/L)	2.16 [1.45–2.87]	2.31 [1.67–3.10]	0.29
Monocytes (10^9^/L)	0.61 [0.44–0.92]	0.73 [0.55–1.18]	0.09
Reticulocytes (10^9^/L)	152 [129–208]	259 [181–334]	**0.0026**
Reticulocytes (%)	4.80 [3.09–8.0]	9.39 [6.65–11.4]	**0.0027**
RET-IRF (%)	33.8 [28.2–39.6]	38.7 [32.8–40.9]	0.16
RET-IRF value (10^9^/L)	48.4 [34.9–87.5]	94.6 [67.8–128]	**0.0048**
RET-He (pg)	30.1 [26.5–32.5]	32.5 [31.2–34.7]	0.066
Reticulocytes/RET-IRF (10^9^/(L*%))	4.94 [3.69–6.61]	6.62 [4.67–9.56]	**0.012**
HbF (%)	3.5 [1.6–6.6]	10 [4.7–14.0]	**0.01**
Hypo-He (%)	6.4 [2.0–12.8]	4.0 [1.6–7.35]	0.34
Hyper-He (%)	0.40 [0.30–0.60]	0.60 [0.40–0.90]	0.067
Micro-R (%)	16.3 [5.7–29.8]	11.3 [5.2–17.2]	0.27
Macro-R (%)	3.6 [3.05–5.05]	4.1 [3.65–7.2]	0.10
LFR (%)	66.3 [61–72.2]	61 [59–65.6]	0.058
MFR (%)	17.1 [15.95–19.75]	19.9 [17.4–20.7]	**0.018**
HFR (%)	16 [11.05–20.7]	19 [13.6–20.9]	0.37
LDH (U/L)	323 [221–422]	410 [264–487]	0.11

*Data are expressed as median and [IQR], n is the total number of patients. RBC, red blood cells; MCV, mean corpuscular volume; MCHC, mean corpuscular hemoglobin concentration; HbF, hemoglobin F; LFR, low fluorescent reticulocytes; MFR, Medium fluorescent reticulocytes; HFR, High fluorescent reticulocytes; VOC, vaso-occlusive crisis. Bold values represent significant differences between the groups*.

### VOC Prediction With Reticulocyte and IRF Measurement

**Figure 2:**
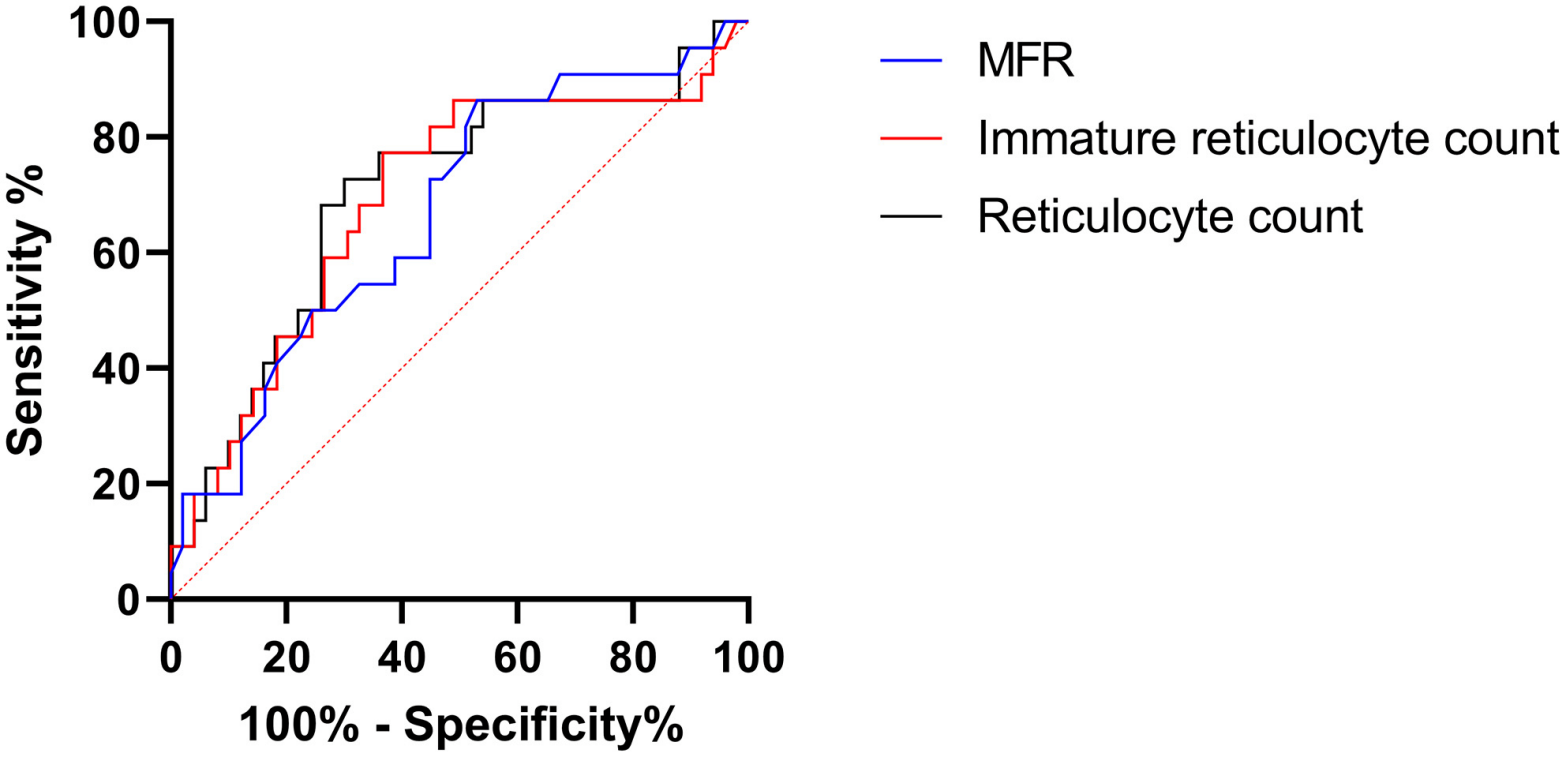
Roc curve of vaso-occlusive crisis prediction by reticulocyte parameters. MFR, medium fluorescent reticulocytes. Immature reticulocyte count: absolute value of immature reticulocytes.

Then, we determined with a ROC curve, the risk of developing VOC: a reticulocyte count of >189.4 10^9^/L (AUC: 0.70, sensitivity: 77.3%, specificity: 64%), an MFR of >19.75% (AUC: 0.67, sensibility: 50%, specificity: 75.5%), and an immature reticulocyte count of >68.6 10^9^/L (AUC: 0.69, sensitivity: 77.3%, specificity: 63.3% respectively) (Figure 2).

**Figure 3:**
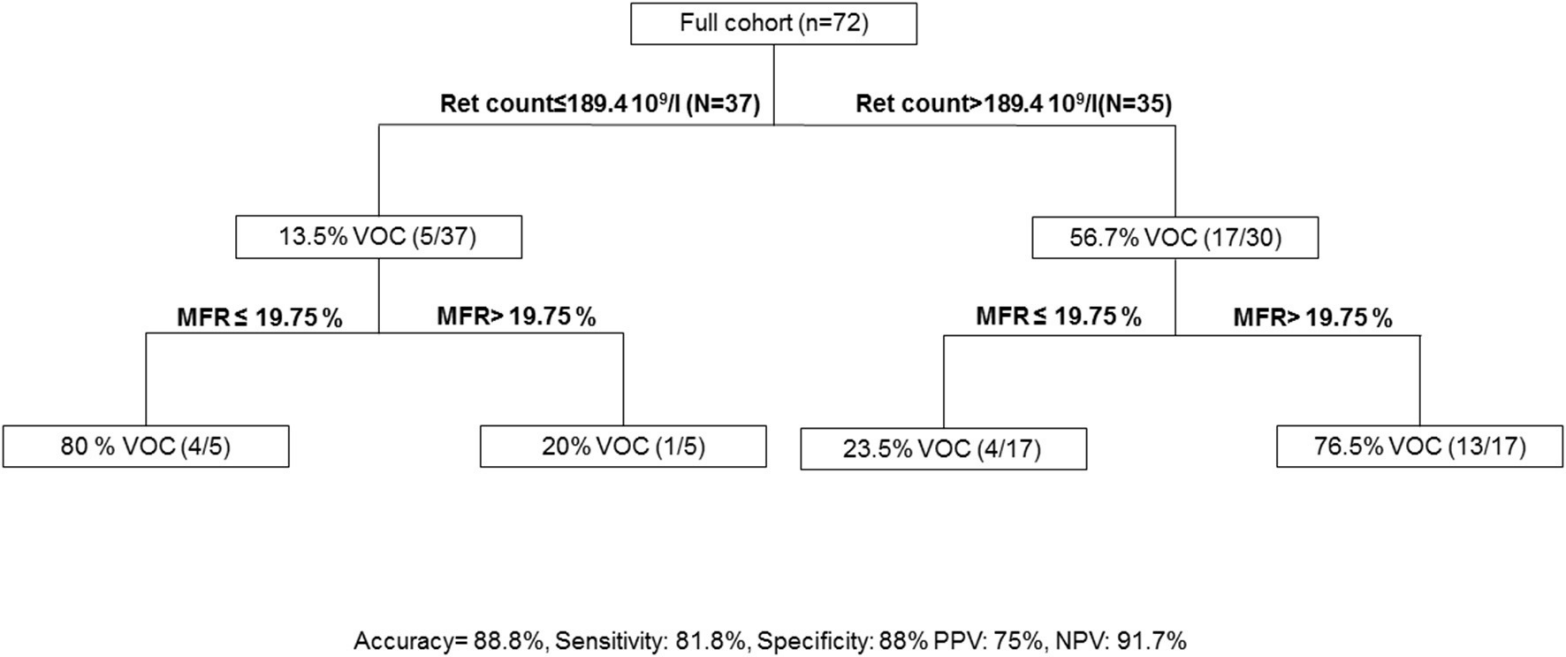
Algorithm to predict vaso-occlusive crisis. MFR, medium fluorescent reticulocytes; Ret count; reticulocyte count; PPV, Positive Predictive Value; NPV, Negative Predictive Value; VOC, Vaso-occlusive crisis.

The association of a reticulocyte count of >189.4 10^9^/L and an MFR of >19.75% presented a sensitivity of 81.8% and a specificity of 88% to predict VOC development in the following year in patients with SCD at steady state (Figure 3).

## Discussion

Our study aimed to demonstrate a correlation between routine laboratory biomarkers of haemolysis as reticulocyte and erythrocyte parameters and the occurrence of VOC in SCD. The association between reticulocyte count and MFR had a good correlation with VOC development.

Sickle cell disease is a lifelong blood disorder affecting ~100,000 people in the United States (11, 12). SCD is a complex genetic disease involving cell adhesion between red blood cells, white blood cells, platelets and endothelial cells, inducing painful VOC (13). Vaso-occlusive crises are characterized by haemolytic anemia, endothelial damage, and potentially life-threatening complications (14). Acute pain is the primary cause of hospitalization in SCD.

Inflammatory syndrome is described in SCD during ACS and VOC (15). Yildrim et al. demonstrated that whole blood count and C-reactive protein at admission could distinguish ACS and VOC (15). In our study, patients with VOC at admission had higher counts of neutrophils and monocytes.

An important disease mechanism involves the release of hemoglobin into the circulation during intravascular haemolysis (14). The release of hemoglobin into the plasma during haemolysis potently inhibits endothelial nitric oxide signaling, leading to endothelial cell dysfunction and nitric oxide resistance (16). Studies have shown correlations between the rate of haemolysis and levels of platelet activation and procoagulant factors in the blood (17). The concentration of LDH has long been recognized as an accurate measure of intravascular haemolysis in SCD (18, 19). In our study, we observed an increase of LDH during VOC. However, this biochemical parameter did not allow to predict VOC development in the following year.

Several routine automated parameters have been developed to evaluate reticulocyte and RBC counts. In SCD, an increase of reticulocyte count is observed in S/S in comparison of S/C (20). In child, S/S patients had most cases of hospital admissions and VOC (21). The reticulocyte count reflects the erythropoietic activity of bone marrow and is thus useful in both diagnosing anemias and monitoring bone marrow response to therapy (22). A lower concentration of RNA in RBC indicates increased maturity, so that immature reticulocytes (HFR and MFR will be classified as a more mature fraction than LFR (10). Immature reticulocyte fraction provides information on bone marrow suppression and recovery of erythropoiesis (23). Erythrocytic parameters has been already used to help the diagnosis of haemoglobinopathy and membranopathy. Automated reticulocyte indices can differentiate hereditary from acquired spherocytosis (4, 6). A previous study, combining reticulocyte count, leukocyte count, and pain in the pelvis and spine, demonstrated a good negative predictive value for ACS risk in SCD (24). Another study demonstrated, with RBC, MCV and MCHC, the possibility of diagnostic orientation in SCD (25). Nivaggioni et al., demonstrated the interest of combining MicroR, MCHC and IRF to differentiate hereditary RBC diseases as spherocytosis and SCD (4). In our study, we observed an association of increased reticulocyte count and increased reticulocyte/IRF during crises. The variation of reticulocyte count was correlated with morbidity in SCD (26).

Laboratory measures can be a powerful supplement to genetic data in predicting morbidity and mortality in SCD (27, 28). Few parameters were associated with VOC prediction. Fetal hemoglobin increase, several β-globin haplotypes and coincidental α-thalassaemia are protective factors of VOC development (26). In children with SCD, ACS was associated with serum level of interleukin 8 and C-reactive protein (29). In adult patients with SCD, a decrease of interleukin 10 in steady state was associated with VOC (AUC ROC curve: 0.688). To our knowledge, our study is the first to evaluate clinical complications in SCD using the reticulocyte parameters. Interestingly, we have shown that a simple score combining reticulocyte count and MFR can be used to predict up to 88.8% of VOC development in the following year. Of note, we observed 17/22 patients with a reticulocyte counts of >189.4 10^9^/L and 13/22 patients with an MFR of >19.75% suggesting the ability of these two parameters to predict clinical outcome.

The ability to predict the phenotype of an individual with sickle cell disease could guide therapeutic decision making. Standard treatment of SCD includes hydroxyurea. In patients with untreated severe SCD, markers of haemolysis were lower in the mild genotype groups regardless of treatment, but they were not lower in severe genotype patients receiving hydroxyurea or chronic transfusions (30). New therapeutic targets are being developed to limit VOC. Voxelotor is an HbS polymerization inhibitor that reversibly binds to hemoglobin to stabilize the oxygenated hemoglobin state (31). Patients treated with voxelotor showed small increases in Hb levels (1 g/dL) and decreased indicators of haemolysis (32). Moreover, a reduced incidence of crises over time was observed. Another treatment is P-selectin target with crizanlizumab (33). P-selectin is expressed on the surface of the endothelium and mediates abnormal rolling and static adhesion of sickle erythrocytes to the vessel surface *in vitro* (34). P-selectin inhibitor crizanlizumab was associated with a significantly lower frequency of sickle cell–related pain crises and appeared to be associated with a low rate of adverse effects (33). The interest of predicting VOC in SCD is to ensure the rapid start of treatment to limit VOC development. Our study has several limitations, including a small sample size. Another limitation was no inclusion of VOC development at home. However, hospitalized VOC are more severe. However, the aim of the study was to build a simple and easy-to-use score to predict VOC development in steady state SCD. Importantly, our score is ready to use in routine laboratory care. The validation of our predictive score is required to ensure the reproducibility of the developed model.

## Conclusion

Based on our findings, a combination of routine laboratory biomarkers, as reticulocyte count, immature reticulocyte count and fluorescent reticulocyte fraction at steady state, could be used to predict VOC development in SCD.

## Data Availability Statement

The original contributions presented in the study are included in the article/Supplementary Material, further inquiries can be directed to the corresponding author.

## Ethics Statement

The studies involving human participants were reviewed and the Institutional Review Board (Rouen University Hospital) approved the study (Authorization protocol number: E2021-78). The patients/participants provided their written informed consent to participate in this study.

## Author Contributions

GF and FK performed the analysis and wrote the manuscript. MG, YB, and AL included patients and critically revised the manuscript and results. VB-S, GB, and SD critically revised the manuscript and results. VL discussed the obtained results and critically revised the manuscript. PB designed the research, analyzed, interpreted the data, and wrote the manuscript. All authors read and approved the final version of the manuscript.

## Conflict of Interest

The authors declare that the research was conducted in the absence of any commercial or financial relationships that could be construed as a potential conflict of interest.

## Publisher's Note

All claims expressed in this article are solely those of the authors and do not necessarily represent those of their affiliated organizations, or those of the publisher, the editors and the reviewers. Any product that may be evaluated in this article, or claim that may be made by its manufacturer, is not guaranteed or endorsed by the publisher.

## Abbreviations

SCD: sickle cell disease
HbS: hemoglobin S
RBC: red blood cells
VOC: vaso occlusive crisis
ACS: acute chest syndrome
VTE: venous thromboembolism
PE: pulmonary embolism
LFR: low fluorescence reticulocytes
MFR: medium fluorescence reticulocytes
HFR: high fluorescence reticulocytes
LDH: lactate dehydrogenase
CBC-O: cell blood count optic
Extended-IPU: Extended Information Processing Unit
Hb: Hemoglobin
MCV: Mean corpuscular volume
MCHC: mean corpuscular hemoglobin concentration
IRF: Immature reticulocyte fraction
Ret-He: Hemoglobin concentration in reticulocytes
%MicroR: percentage of microcytic erythrocytes
%Macro-R: percentage of macrocytic erythrocytes
IQR: interquartile ranges
ROC: Receiver operating characteristic
Hypo-He: hypo-hemoglobin content in erythrocytes
Hyper-He: hyper-hemoglobin content in erythrocytes
Ret-He: Hemoglobin concentration in reticulocytes.
