# Supplementary material for: Assessment of Reticulocyte and Erythrocyte Parameters From Automated Blood Counts in Vaso-Occlusive Crisis on Sickle Cell Disease
Source: Front Med (Lausanne). 2022 Apr 13;9:858911. doi: 10.3389/fmed.2022.858911 (PMC9044919; doi:10.3389/fmed.2022.858911)
Supplement: Supplementary file 1 [file Table_1.DOCX]

|  | **Without VOC in a year** | | | **With VOC in a year** | | |
| --- | --- | --- | --- | --- | --- | --- |
|  | **S/S-Sβ^0^**  **n=18** | **S/Sα^3.7^**  **n=16** | **S/C-Sβ^+^**  **n=16** | **S/S-Sβ^0^**  **n=14** | **S/Sα^3.7^**  **n=5** | **S/C-Sβ^+^**  **n=3** |
| RBC (10^12^/L) | 2.86 [2.26-3.22] | 3.14 [2.83-4.17] | 4.34 [3.8-4.8] | **2.63 [2.37-3.09] ^b,c^** | 3.01 [2.79-3.74] | 3.89 [3.76-3.9] |
| Haemoglobin (g/dL) | **8.5 [7.8-9.65] ^d^** | 8.9 [8.1-10.1] | 10.5 [10.1-10.9] | **8.0 [7.8-9.8] ^c,e^** | 8.2 [7.6-9.4] | 11.4 [10.5-11.6] |
| Haematocrit (%) | 24 [22-28] | 27.5 [23.5-30.8] | 31 [29-32] | **23 [22-28.5] ^a,b^** | 24 [23-27] | 30 [29-32] |
| Reticulocytes (10^9^/L) | 161 [136-296] | 193 [136-286] | 140 [109-151] | **292 [245-371] ^a,b^** | 232 [137-319] | 108 [108-152] |
| RET-IRF (%) | 34.2 [32.0-40.8] | 35.7 [32.7-45.6] | 32.1 [25.4-37.0] | 38.7 [34.6-41.4] | 40.4 [30.5-41.5] | 25.5 [23.3-35.1] |
| RET-IRF value (10^9^/L) | 56.7 [41.8-110.4] | 87.9 [44.6-118.1] | 40.1 [30.8-59.6] | **115 [81.4-136] ^a,b^** | 93.7 [47.8-130] | 27.5 [25-53.4] |
| RET-He (pg) | 35.1 [27.7-38.4] | 29.9 [29.4-31.7] | 28.6 [24.0-31.5] | **34.2 [31.8-37.4] ] ^a,b^** | 31.4 [23.7-32.3] | 31.9 [31.4-34.6] |
| Reticulocytes/RET-IRF (10^9^/(L*%) | 2.92 [2.46-3.13] | 2.79 [2.19-3.06] | 3.12 [2.70-3.94] | 2.59 [2.41-2.89] | 2.48 [2.41-3.61] | 3.92 [2.85-4.3] |
| Hypo-He (%) | 5.5 [0.65-17.3] | 6.1 [3.63-13.2] | 6.4 [1.7-12.3] | 5 [1.7-10] | 5.4 [3.3-27.1] | 1.2 [0.6-1.7] |
| Hyper-He (%) | 0.7 [0.3-8.5] | 0.35 [0.3-0.53] | 0.4 [0.3-0.5] | 0.8 [0.5-4.4] | 0.4 [0.2-0.55 | 0.6 [0.4-0.6] |
| Micro-R (%) | 9.5 [0.9-20.7] | 13.6 [6.8-29.6] | 24.7 [12.9-41.2] | 11.3 [3.5-16.7] | 11.9 [8.134.5] | 7.0 [6.5-17.1] |
| Macro-R (%) | 5.6 [3.3-15.4] | 3.9 [3.2-4.7] | 3.2 [2.5-3.5] | 5.9 [3.9-13.1] | 4.1 [2.6-4.3] | 3.5 [3.3-3.6] |
| LFR (%) | 65.8 [59.3-68.1] | 64.5 [55.8-70.6] | 68.0 [63.0-74.6] | 61 [58.3-64.6] | 59.6 [58.5-69.6] | 74.5 [64.9-76.7] |
| MFR (%) | 16.7 [15.2-18.5] | 18.8 [16.3-20.2] | 18.4 [16.0-19.5] | 19.9 [17.6-21.2] | 19.9 [16.6-21.6] | 17.1 [15.120.3] |
| HFR (%) | 18.4 [15.5-21.2] | 17.5 [12.4-24.8] | 13.3 [9-19.35] | 19.8 [16.0-23.5] | 19.7 [13.8-20.6] | 8.4 [8.2-14.8] |

**Supplemental table 1.** Data are expressed as median and [IQR], n is the total number of patients. RBC: red blood cells. LFR: low fluorescent reticulocytes. MFR: Medium fluorescent reticulocytes. HFR: High fluorescent reticulocytes. VOC: vaso-occlusive crisis.

**^a^** Indicates a significant difference between S/C-S/β^+^ and S/S-S/β^0^ in VOC

**^b^** Indicates a significant difference between S/S-S/β^0^ in VOC and S/C-S/β^+^ in VOC

^c^ Indicates a significant difference between S/S-S/β^0^  and S/C-S/β^+^

^d^ Indicates a significant difference between S/S-S/β^0^ and S/C-S/β^+^

^e^ Indicates a significant difference between S/C-S/β^+^ in VOC and S/Sα^3.7^ in VOC
